# Performance Analysis of a Microfluidic Pump Based on Combined Actuation of the Piezoelectric Effect and Liquid Crystal Backflow Effect

**DOI:** 10.3390/mi10090584

**Published:** 2019-08-31

**Authors:** Yanfang Guan

**Affiliations:** School of Electromechanical Engineering, Henan University of Technology, Zhengzhou 450001, China; yguan@haut.edu.cn

**Keywords:** piezoelectric transducer, liquid crystal, combined driving mode, micropump

## Abstract

A novel combined actuation method based on the piezoelectric effect and liquid crystal backflow effect is proposed in this paper. The coupling mechanism of a piezoelectric transducer (PZT) and liquid crystal (LC) in a combined driving mode is analyzed, and the governing equations of electromechanical coupling based on inverse piezoelectric effect and the classical Leslie–Ericksen backflow equation are modified under combined driving method. The new multifield coupling dynamic equations for numerical analysis is established. Experimentally, a sandwiched micropump was manufactured and sealed with wet etching technology on a glass wafer. A testing platform was built to analyze the particles motion and the flow rates were measured with both single PZT or LC actuation and combined actuation. Comparing the results of the numerical analysis and experimental testing of the flow rate and LC molecule motion under different driving voltages and frequencies, the performance of the PZT/LC combined driving is found to be superior to that of the single driving mode (PZT or LC driving) under the same driving conditions. Moreover, the new combined driving mode overcome the disadvantages of single driving mode and enhance the driving efficiency significantly. The simulation results are in good agreement with the experimental data. The maximum flow rate of the micropump achieved was 4.494 μL/min with combined driving method.

## 1. Introduction

The concept of micro electromechanical system (MEMS) originated in the late 1970s, stemming from using the piezoelectric effect of semiconductor Si for controlling microsystems. Despite many years of development, it is still a frontier research field involving many complex multi-physical problems that require interdisciplinary approaches [1,2,3]. As an important branch of MEMS, microfluidic actuation and control systems are widely used in drug delivery, biosensing, and electronics cooling due to its small size, low power consumption, high control precision, and fast response [4,5,6]. Generally speaking, microfluidic actuation methods include mechanical and non-mechanical modes, and the latter has the advantages of simple architecture due to no moving parts, ease of miniaturization and integration, flexible driving method, high reliability, and long service life [7,8,9]. As a result, unlike MEMS, the non-mechanical actuation mode has become popular in microfluidic systems [10,11,12,13,14].

The commonly used microfluidic non-mechanical actuation methods mainly include electroosmotic [15,16,17], magnetic [18,19], optical [20,21,22], thermal [23,24], and piezoelectric [25,26,27]. As a common microfluidic driving method, piezoelectric actuation has been widely used due to its low cost, easy operation, and high efficiency [28,29]. Kasai et al. constructed a high-speed-on-chip micropump that could sort cells with synchronized actuation of a piezo-driven dual membrane pump [25]. Munas et al. presented a sandwiched micropump with a PZT actuating on polymethylmethacrylate (PMMA) sheets [26]. The finite element method (FEM) analysis and experiment were carried out. Demori et al. presented and tested fluid and particle manipulation by two piezoelectric actuators [30]. They demonstrated that fluid mixing and controlled positioning of dispersed particles are possible. However, the high driving voltage and frequency, low vibration amplitude, and difficult self-priming limited the usage of piezoelectric actuators for microfluidics applications [31,32].

On the other hand, liquid crystal backflow has emerged as a new actuation method in recent years because of its easy control in topological structure, and the rotation and movement characteristics of the microdroplet [33]. The liquid crystal phase is a liquid formed by rod/disk-shaped organic molecules. Guo et al. proved the transfer of physical and chemical properties with cholesteric liquid crystal microdroplets by adding a chiral dopant into the solvent. The results provided an easy, cheap, and reliable way to manipulate the microdroplets in a microfluidic system [33]. Yoshitaka et al. studied the microparticles’ movement performance of the liquid crystal bidirectionally using the backflow effect and the electrostatic force [34]. Priest et al. investigated the microfluidic principles, and the potential for chip-based multilayer assembly for the synthesis of polymer microcapsules by dispersing liquid crystal molecules in an aqueous continuous phase [35]. Deng et al. developed an artificial molecule transfer technology, in which flow speed could be controlled and switchable by tuning the molecular solubility in a continuous phase [36]. However, the low driving force and small motion distance limited the usage of the liquid crystal for microfluidic actuation [37].

Since each single actuation mode has its disadvantages, it is often necessary to combine different actuation methods to effectively solve the problems existing in single methods, such as strict requirements on particles, high power consumption, and low efficiency [38,39,40]. In the past, researchers have explored the combination of piezoelectric actuation to other methods. For example, Li et al. demonstrated a precise stepping drive motor with piezoelectric/electrorheological characteristics [41]. Zhang et al. analyzed in detail the influencing factors of the dispensing quality of a piezoelectric/magnetic hybrid non-contact injection point adhesive machine [42]. Zheng et al. collected and sorted the time-averaged energy collector based on piezoelectric effect [43]. Kim et al. studied in detail the influence of driving voltage and jet frequency of piezoelectric hybrid inkjet printing technology on droplet volume [44]. Aziz et al. developed a new type of piezoelectric/thermoelectric hybrid energy collector based on piezoelectric/thermoelectric hybrid energy collector [45]. Hamed et al. studied the electrostatic characteristics and the driving voltage model of a piezoelectric/electrostatic-driven micro-switch [46]. Zhou et al. analyzed the key parameters of piezoelectric-driven diaphragm microdroplet injection technology [47]. The empirical formula of injection velocity is given, and a micro injection test system is established. In all these studies, piezoelectric actuation serves the main role in the devices and the working fluid never played any real roles.

In this study, we proposed a novel combined microfluidic driving method based on a liquid crystal and piezoelectric transducer. The mechanism of the novel hybrid actuation combines the piezoelectric effect and liquid crystal backflow effect, where the liquid phase also plays a significant role in actuation. The novel hybrid method is expected to achieve higher net motion of flow in microfluidics under low driving voltage and capacity. In addition, the new method will overcome the low self-priming characteristic of the PZT driving, expanding the application scope to most types of microparticles. Moreover, the coupling numerical model and micropump under LC/PZT combined driving have been established and fabricated, respectively. As a proof of concept, the results from numerical analysis and experiment both show the combined model is feasible in the xbiomedical field.

## 2. Theory and Methods

**Figure 1:**
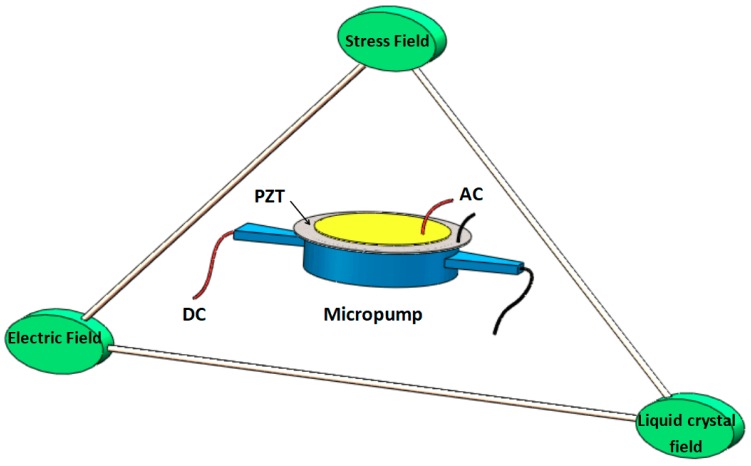
The schematic of the multifield coupling principal of liquid–solid–stress with LC and PZT combined driving by alternating electric (AC) and direct electric (DC) power supplies.

The working principle of combined PZT/LC driving mode is more complex than that of single drive. The driving process involves an electric field, stress (or mechanical) field, and LC flow field. In this section, the coupling mechanism analysis will start with the establishment of a multi-field coupling numerical model that includes the electromechanical coupling, fluid-solid coupling, and mechanical-liquid coupling concerning the constitution equation of the electric, stress, and LC flow fields. Furthermore, the numerical analysis equations of multi-field coupling driven by the combination method are revised and established. The analysis of combination drive theory is shown in Figure 1.

### 2.1. Combined Driving Mechanism

**Figure 2:**
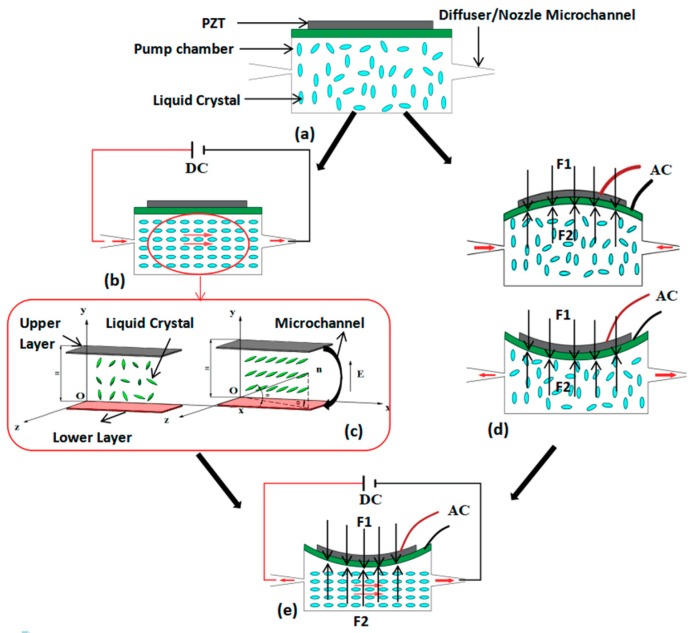
Working principal of the combined driving of piezoelectric transducer and liquid crystal. (**a**) No power supply. (**b**) AC supply. (**c**) The backflow effect of the LC with the AC power supply. (**d**) Inverse piezoelectric effect of the PZT with the DC supply and the supply mode and pump mode for the micropump. (**e**) The net flow of the LC with both AC and DC power supplies.

The working principle of combined PZT/LC actuation is shown in Figure 2. In the beginning, liquid crystal molecules are disorganized and randomly distributed in the microchannel, as shown in Figure 2a. When a DC field is applied, the molecules rotate and align with the electric field, resulting in a so-called backflow effect, as shown in Figure 2b. By controlling the direction of the applied electric field, the liquid crystal produces a directional flow (Figure 2c). On the other hand, when an AC signal is applied to the upper and lower surfaces of the piezoelectric actuator, the piezoelectric transducer will produce an inverse piezoelectric effect, which will in turn produce Z-direction vibration, and the change of the deformation will cause the volume change of the micro-pump cavity connected to the piezoelectric actuator. As shown in Figure 2d,e, the inlet flow can be transported directionally by unsymmetrical arrangement of the diffuser/nozzle microchannel. The single driving mode (PZT or LC) and the combined driving mode (PZT and LC) can be realized by controlling the number of applied electric fields in the process of this work. In the combined working process, the inverse piezoelectric effect of the piezoelectric actuator and the backflow effect of the liquid crystal are used. The advantages of the great piezoelectric driving force and the arbitrary change of the driving direction of the liquid crystal are used. It makes up for the shortcomings of low driving force of liquid crystal backflow. The function of fluid directional transport can be realized by reasonable arrangement of micro-channel structure, which constitutes a real microfluidic PZT/LC combination driving mode. In order to provide practical feasibility for the microfluidic combination drive, the performance of the micro-pump will be greatly improved.

### 2.2. The Establishment of Multi-Field Coupling Numerical Model

The flow medium of 5CB filamentous nematic liquid crystal is used in this study, which is different from the ordinary continuous fluid, in that the molecule is long rod and has a direction vector; that is to say, the rotation of the LC molecule occur during the net flow process. The rotation is described by the direction vector n, shown in Figure 2c. For convenient calculation, the size of n is usually taken as 1. In this study, the flow process of LC under electric field can be regarded as incompressible continuous fluid according to the traditional Leslie–Ericksen theory [48,49]. Additionally, the flow dynamic characteristics of liquid crystal molecule under external electric field driving are all expressed by velocity field. Moreover, the orientation field of liquid crystal molecules is determined by the direction vector n, and the change of the direction vector n is determined by the velocity gradient.

Therefore, the control equations of liquid crystal flow under the action of the DC electric field (all the center points in the equation represent the point multiplication calculation) includes the continuity equation, the momentum equation, and the angular momentum equation of the liquid crystal molecule:

(1) ∇·v=0

(2) $$\rho \left\{\frac{\partial v}{\partial t}+\left(v\cdot \nabla \right)v\right\}=F+\nabla \cdot \left(-pI+\tau \right)$$

(3) $$n\times \left\{G+\frac{\partial F}{\partial n}-\nabla \cdot \left(\frac{\partial F}{\partial \nabla n}\right)+\lambda_{1}N+\lambda_{2}A\cdot n\right\}=0$$

where ∇ is the Hamilton operator; ν is the velocity vector; ρ is the density of the liquid crystal; *t* is the time; F is the force; p is the pressure; I is the unit vector; τ is the deviation stress vector from constitutive equation; n is the direction vector; G is the stress tensor; N is the exponential vector and the relative angular velocity vector of the LC molecule; and A is the deformation speed tensor.

In Equation (2), the external force *F* caused by the electric field is:

(4) $$F=\left[\left(\varepsilon_{⊥}E+\varepsilon_{a}\left(n\cdot E\right)n\cdot \nabla \right)\right]E$$

where E is the electric field intensity; $\varepsilon_{a}$ is the dielectric anisotropy of the liquid crystal, which can be expressed as the difference between the dielectric constant of the liquid crystal $\varepsilon_{∥}$ that is parallel to the direction vector  n  and the dielectric constant of the liquid crystal $\varepsilon_{⊥}$ that is perpendicular to direction vector  n. The equation is shown as follows:

(5) $$\varepsilon_{a}=\varepsilon_{∥}-\varepsilon_{⊥}$$

The constitutive equation of liquid crystal is different from that of isotropic fluid. The rotation of the direction vector n relative to fluid flow direction must be taken into account. The constitutive equation of nematic liquid crystal proposed by Leslie is shown as follows:

(6) $$\tau =\alpha_{1}nnn\cdot A\cdot n+\alpha_{2}nN+\alpha_{3}Nn+\alpha_{4}A+\alpha_{5}nn\cdot A+\alpha_{6}A\cdot \mathrm{nn}-\frac{\partial F}{\partial \nabla n}\cdot {\left(\nabla n\right)}^{T}$$

(7) $$N=\frac{dn}{dt}-\Omega \cdot n$$

(8) $$\Omega =\frac{1}{2}\left\{{\left(\nabla v\right)}^{T}-\nabla v\right\}$$

(9) $$A=\frac{1}{2}\left\{{\left(\nabla v\right)}^{T}+\nabla v\right\}$$

where $\alpha_{i}\left(i=1,\cdots ,6\right)$ is the Leslie viscosity coefficient, $\alpha_{1}\sim \alpha_{6}$ equal to 0 Pa∙s, −0.086 Pa∙s, −0.004 Pa∙s, 0.089 Pa∙s, 0.059 Pa∙s, −0.031 Pa∙s, respectively. $\lambda_{1},\;\lambda_{2}$ in Equation (3) can be expressed as: $\lambda_{1}=\alpha_{3}-\alpha_{2}$, $\lambda_{2}=\alpha_{3}+\alpha_{2}$, respectively. G is the volume force tensor that represents the electric field acts on the direction vector n, and its expression is shown as follows:

(10) $$G=\varepsilon_{a}\left(n\cdot E\right)E$$

The vector expressions of the speed, direction vector n and the electric field vector in the coordinate system of Figure 2c are shown as follows:

(11) $$v={\left(u,0,w\right)}^{T}$$

(12) $$n={\left(\cos\emptyset \sin\theta ,\;\sin\emptyset ,\cos\emptyset \cos\theta \right)}^{T}$$

(13) $$E={\left(0,E,0\right)}^{T}$$

The F in Equation (3) reflects the free energy density produced by the spatial deformation of liquid crystal (that is different from the force F in Equation (2)). According to the Frank elastic deformation theory, there are:

(14) $$2F=K_{1}{\left(\nabla \cdot n\;\right)}^{2}+K_{2}{\left(n\cdot \nabla \times n\;\right)}^{2}+K_{3}{\left|n\times \nabla \times n\right|}^{2}$$

Among them, $K_{1},\;K_{2},\;K_{3}$ is the elastic coefficient of liquid crystal expansion, distortion, and bending. The material properties of the liquid crystal are shown in Table 1.

The coupling analysis of the piezoelectric transducer was carried out between the piezoelectric ceramic and copper layer. The electrode layer and adhesive layer are very thin and were neglected. The upper surface of the piezoelectric body is generally a free surface, and the lower surface is in contact with the copper layer. Hence, coupling analysis was adopted using an applied load at the lower surface of the piezoelectric material. The electrical-mechanical coupling equation for the material of PZT-5H is [50]:

(15) σ=Cε−eE

where σ is the mechanical stress tensor. C is the elastic stiffness constant. ε is the mechanical strain tensor. e is the piezoelectric continuous tensor. E is electric vector. The material parameters of the PZT are shown in Table 2 for calculating.

### 2.3. Revising of Movement Equation under Combined Driving Mode

In the combined driving mode, the external force term at the right end of the Equation (2) mainly considers three effects, that are, the external force vector of the liquid crystal molecule caused by the double electric field under the combined driving mode. The force generated by the forced vibration of the piezoelectric oscillator is transferred directly to the fluid. The fluid also has a reaction force on the piezoelectric oscillator, and the interaction force between the liquid crystal field and the piezoelectric actuator. Moreover, in the combined driving mode, due to the special structure and asymmetric arrangement of the diffuser/nozzle microchannel, the pressure difference between the inlet and outlet of liquid crystal flow cannot be approximated to zero in the traditional L–E theory. Based on the above three points, the external force F  and pressure P in the movement Equation (2) need to be modified respectively.

The detailed methods for revising are shown as follows: the force generated by the forced vibration of the piezoelectric oscillator $F_{1}$, the reaction force of the fluid on the piezoelectric oscillator $F_{2}$, as shown in Figure 2d,e, so $F^{\prime }$ is added to the Equation (2) to replace the force F, which represent the force at the interface between the liquid crystal flow field and the piezoelectric actuator. The solution method can be carried out by simultaneously solving Equations (2) and (15). The pressure difference at inlet and outlet ends of liquid crystal flow in Equation (2) can be calculated by $P^{\prime }=\frac{64}{Re}\frac{l}{d}\frac{\rho v^{2}}{2}$. Based on the above two points, the new revised movement equation under combined driving mode is shown as follows:

(16) $$\rho \left\{\frac{\partial v}{\partial t}+\left(v\cdot \nabla \right)v\right\}=F^{\prime }+\nabla \cdot \left(-P^{\prime }I+\tau \right)$$

All the above equations are combined to form the equations of multi-field coupling numerical analysis under combined PZT/LC actuation driving.

### 2.4. Initial and Boundary Conditions for Numerical Analysis

The Reynolds number of the flow field in the piezoelectric/liquid crystal combined actuation system is small, and the density and temperature of the liquid crystal fluid are assumed to be fixed values, so the flow of the liquid crystal flow field can be approximately laminar flow of the incompressible fluid at low Reynolds number. The density, dielectric coefficient, refraction coefficient, elastic constant, and viscosity coefficient of liquid crystal materials are shown in Table 1 for calculating and analyzing.

The tetrahedral mesh and three-dimensional model is used in the numerical calculation process. The entrance and exit boundary conditions are set on both sides of the model, and the around is wall boundary. The physical fields involved in the calculation include laminar flow, solid mechanics (piezoelectric), electrostatic and particle tracking modules. The multi-physical fields coupling module include fluid-solid coupling, electro-liquid coupling and mechanical-electrical coupling modules.

The sinusoidal AC voltage signal applied to the upper and lower surfaces of the piezoelectric oscillator are shown as follows:

(17) $$U_{1}=U_{0}\mathrm{Sin}\left(2\pi ft\right)$$

where $U_{0}\;$ is the initial voltage. The initial value of the AC electric field driven by piezoelectric actuation is $U_{1}=30\;\mathrm{Vpp},\;50\;\mathrm{Vpp},\;70\;\mathrm{Vpp}$, and the initial value of the DC electric field driven by the liquid crystal is $U_{2}=30\;V,\;50\;V,\;70\;V$, respectively.

The periphery of the piezoelectric oscillator is set as the clamping boundary in the electric-mechanical coupling module, and the governing equations are shown as follows:

(18) $$\frac{\partial^{2}W}{\partial x^{2}}=\frac{\partial^{2}W}{\partial y^{2}}=\frac{\partial^{2}W}{\partial z^{2}}=0$$

(19) $$\frac{\partial W}{\partial t}=0$$

where the displacements in the direction of *x*, *y*, and *z* is zero in the round periphery.

The initial inclined angle of liquid crystal is $\theta \left(0\right)=\theta \left(H\right)=1^{{}^{\circ}}$, and the initial reverse angle is $\phi =0^{{}^{\circ}}$, as shown in Figure 2c. Thus, Equations (1)–(20) form the numerical equation set under the combined driving. In the numerical calculation, the second-order central difference method is used in space and the second-order Runge–Kutta method is used in time.

### 2.5. Structural Design

**Figure 3:**
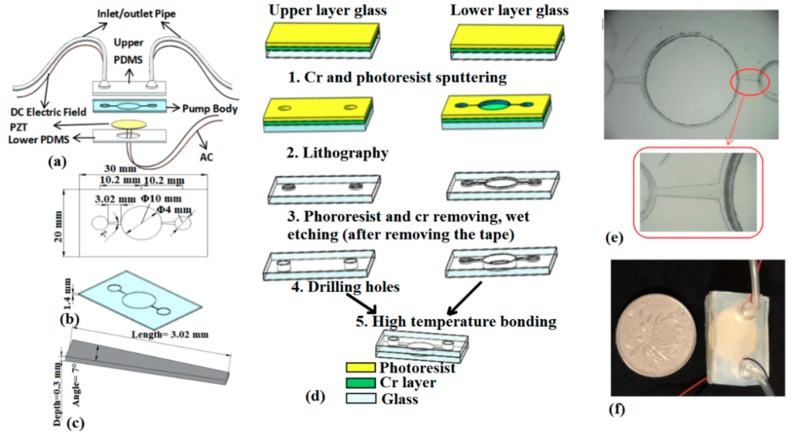
Structural design and manufacture of the micropump with glass wafer. (**a**) 3D structure of the micropump with PZT/LC combined driving. (**b**) The dimensions of the pump body. (**c**) The dimension of the microchannel. (**d**) The fabrication process of the microchannels and chambers by wet etching on glass wafer. (**e**) The microphotograph of the microchannel and pump chambers after wet etching. (**f**) The photograph of the micropump after packaging.

The three-dimensional structure diagram of a sandwiched micropump is shown in Figure 3a. The main structure includes a glass-based pump body, a piezoelectric actuator and upper and lower PDMS cover body, in which the upper PDMS cover body is consolidated with an inlet and outlet pipe and a DC power supply application channel. The structure of the glass-based pump body is shown in Figure 3b. The size of the substrate is 10 mm, the diameter of the pump cavity is 10 mm, and the diameter of the inlet and outlet pipe is 4 mm. The structure size of diffuser/nozzle microchannel is shown in Figure 3c, the length is 3.02 mm, and the expansion angle is 7° (this value has been optimized in the literature [29]).

The diffuser/nozzle microchannel, inlet/outlet pump chamber were fabricated by wet etching process [51,52] of glass-based pump body, and the process is shown in Figure 3d. Firstly, the upper and lower layers of the glass were coated with a chromium layer (Cr) and photoresist (step 1) after cleaning, and then the pump chamber, microchannel, and inlet/outlet pump chamber were processed by a JKG-2A lithography machine (Shanghai Xueze Optical Instrument Co., LTD, Shanghai, China) under the protection of photoresist (step 2). After lithography, photoresist was removed with NaOH solution. The two layers of glass matrix were dried in vacuum drying box to remove the clean Cr layer (step 3). Then the glass wafer was coated with the normal tape exclude the central pump chamber, inlet/outlet pump chamber and microchannels. The double layers glass is immersed in the solution made of NH_4_F + HNO_3_ + HF according to a certain proportion, corroded for 150 min. Then, the depth of 300 microns (step 3) is obtained. After removing the tape and residue the glass wafer is located in the deionized water and vacuum drying box. Finally, the upper and lower layers of glass are placed on the drilling machine, and the pump chamber from the backside, the inlet and outlet holes from the front side are drilled through the whole glass thickness (step 4), but the inlet and outlet holes on the front of the lower glass remain unchanged. Moreover, in order to seal the microchannel on the front side of the lower glass, the back of the upper glass is aligned to the front of the lower glass by high temperature bonding method, which includes first heating in a 540 °C oven for 3 h, and then cooled down to room temperature. The two layers of glass are tightly bonded at low temperature (step 5). Figure 3e is the microchannel structure after etching.

At the end, in order to realize the complete pumping function, the upper and lower polydimethylsiloxane (PDMS) were fabricated, in which the upper PDMS was designed with an inlet and outlet pipe, which corresponds to the inlet and outlet holes on the front of the upper glass. A circular hole is opened in the middle of the lower PDMS to facilitate the fixing of the PZT, for efficient vibration actuation of the PZT. For sealing the micropump entirely, double-sided tape (3M, 4377-50, Shanghai, China) is used to fix the PZT on the backside of the pump chamber firstly, and then two layers of PDMS after surface modification by UV light were covered on the front and back sides of the glass wafer to form an irreversible bond. The periphery of the PZT was fixed on the back side by 3M and PDMS tightly. The packaged micropump structure is shown in Figure 3f and is the same size as a coin.

### 2.6. Layout of Experiment

**Figure 4:**
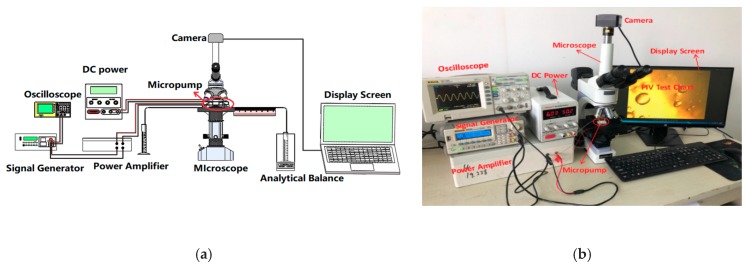
Layout of the experiment setup and control system. (**a**) Schematic of the flow rate and flow field of the micropump with different driving methods. (**b**) Photograph of the performance testing system of the whole micropump with LC/PZT driving methods.

The experimental setup used to study the performance of PZT/LC combined driving of a micropump mainly includes two parts: the actuation module (piezoelectric and liquid crystal) and the flow characterization module (flow rate measurement), as shown in Figure 4. The instruments used in the experiment mainly include a signal generator (RIGOL DG1022, RIGOL Technologies, Beijing, China), a home-made power amplifier, a DC power supply (MAISEN DC POWER SUPPLY, Shenzhen Ever Good Electronic Co., Ltd., Shenzhen, China), an oscilloscope (RIGOL DS1102E, RIGOL Technologies, Beijing, China), an analytical balance (MS-TS, Mettler Toledo, Columbus, OH, USA), a fluorescence microscope (Obvious Ltd., Co., Beijing, China), a CCD high-speed camera (CCD, Cube3, Mikrotron, Unterschleissheim, Germany), etc.

Two types of experiments were performed: Single piezoelectric or liquid crystal actuation and combined actuation. The driving voltage and frequency are the same as those in the simulation analysis. Before the experimental test, the output of the signal generator is connected to the input of the amplifier, and then it is input to the piezoelectric oscillator, which is used as the power supply of the piezoelectric actuator. At the same time, the output of DC power supply is connected to the inlet and outlet wire in Figure 3a,f, which is used as the power supply of the liquid crystal backflow driving. Then the inlet pipe, outlet pipe and the micropump body is placed on the same plane under the microscope to cancel the pressure difference between the inlet and outlet pipes, as shown in Figure 4b. Before the actual flow field test, the sealing performance of the micropump is first tested, and the driving modules are checked to ensure that the functions of the entire test system are intact. The piezoelectric and liquid crystal driving voltages and frequencies can be controlled by adjusting the DC power supply and the signal generator. The LC solution is injected into the inlet pipe after being mixed at 15:1 with polystyrene fluorescent particles (PS, 2005A, Thermo Fisher Scientific, Shanghai, China) for easy observing the flow field. The flow field visualization and characterization was performed using the microfluidic field platform with the help of PS particles. At the outlet of the micropump, the liquid crystal liquid is collected for a period of time (ΔT = 3 min), and then the flow rate of the micropump is calculated by weighing method as follows:

(20) $$\emptyset =\frac{W\times 10^{6}}{\rho \Delta T}\left(\mu L/\min\right)$$

where ∅ is the flow rate, W is the weight of liquid in the outlet reservoir (in g), and ρ is the liquid density.

## 3. Results and Discussion

### 3.1. Analysis of Numerical Results

**Figure 5:**
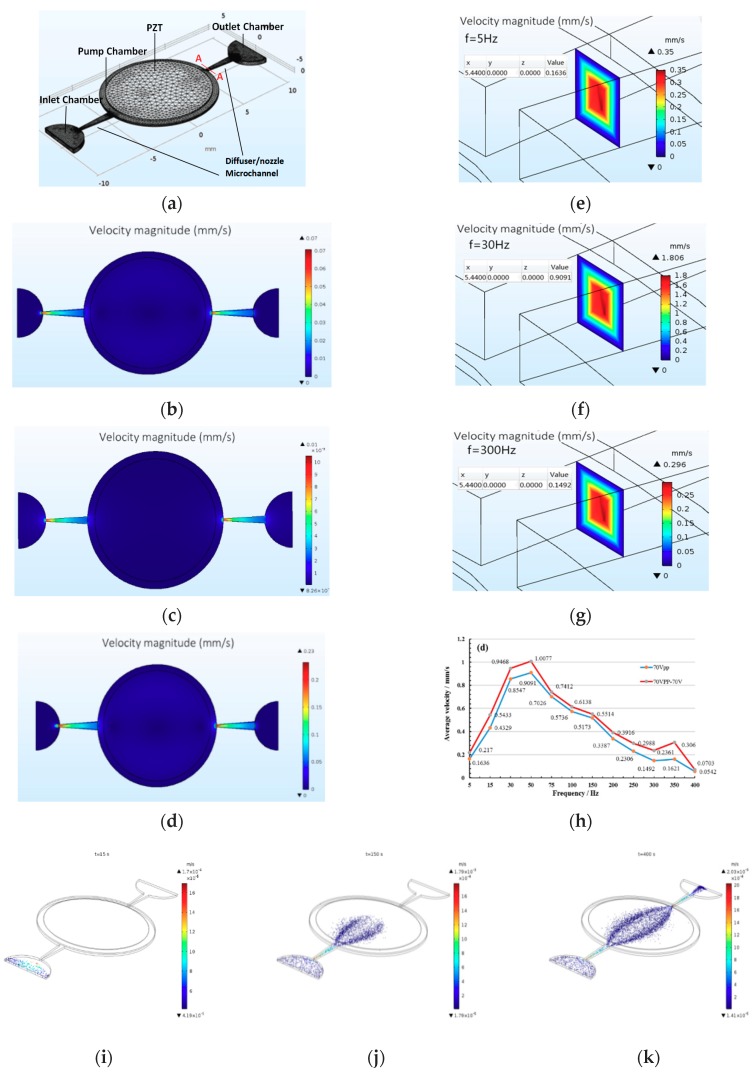
The FEA model and numerical analysis results of the whole micropump. (**a**) FEA model of the micropump with combined LC/PZT driving. (**b**–**d**) The velocity contours of pump chamber and the microchannel under single PZT driving, single LC driving and combined driving. (**e**–**g**) The velocity contours of the A–A cross-section under 5 Hz, 30 Hz, and 300 Hz driving frequencies. (**h**) The average velocity curve vs. frequency. (**i**–**k**) The flow trajectory of liquid crystal particles with the DC power supply at 15 s, 150 s, and 400 s.

The numerical analysis has been conducted with single PZT, single LC, and combined driving in three kinds of driving voltages. For PZT driving, the driving frequency is from 5 Hz to 400 Hz. The flow field distribution of single PZT driving, liquid crystal driving, and combined driving are shown in Figure 5 with 70 Vpp and 70 V driving voltages. The cross-section shown in Figure 5b–d is taken from the central section along the Z direction of the three-dimensional model in Figure 5a. The velocity magnitude increases with the combined driving, PZT driving and LC driving in sequence from Figure 5b–d.

In order to obtain the detailed relationship between the velocity of the liquid crystal and the driving voltage, frequency, a cross-section A–A (cross-section parallel to the y–z plane, x = 5.4400 mm) is used as a reference in Figure 5a. The velocity cloud diagrams have been adopted to study the changing trend between different driving method, voltage, and frequency combined. For convenience, just part of velocity diagrams (under 70 Vpp and 70 V combination) of A–A cross-section are selected and shown in Figure 5e–g.

The velocity distributions of liquid crystal flow of A–A cross-section under 70 Vpp of driving voltage, 5 Hz, 30 Hz, and 300 Hz of driving frequency with PZT driving, and 70 V of driving voltage under LC driving are shown in Figure 5e–g. The maximum, minimum, and average velocity of the liquid crystal flow can be clearly seen from Figure 5e–g. The velocity value of the liquid crystal is greatest under the 30 Hz driving frequency. For ease of comparison and analysis the changing trend and the average velocity via the whole driving frequencies are collected and shown in Figure 5h under single PZT driving and PZT/LC combined driving. It can be seen that the mean velocity increases first and then decreases with the increase of the frequency under the single piezoelectric drive mode and the combined drive mode from the average velocity diagram of section A–A in Figure 5h. There is a maximum value the piezoelectric driving frequency at 50 Hz. The average velocity value is greater under combined driving than that of single PZT driving. The results preliminarily prove that the combined driving performance is superior.

**Figure 6:**
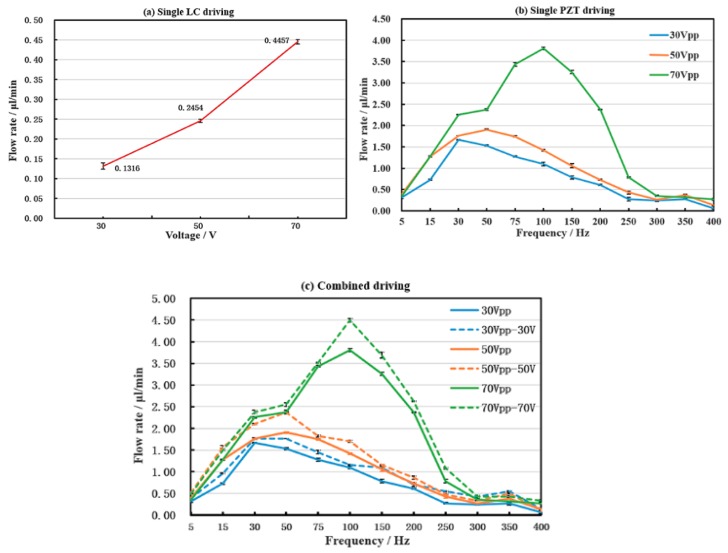
The flow rate of the micropump with (**a**) single LC driving, (**b**) single PZT driving, and (**c**) combined driving.

The flow trajectories of liquid crystal are shown in Figure 5i–k in the micropump under 70 V of combined driving voltage after adding particle tracing module during numerical analysis for obtaining detailed knowledge of the flow conditions inside of the micropump. The ratio of the polystyrene (PS) fluorescent particles and liquid crystal is 1:15, and the diameter of the fluorescent particles is 0.5 µm. As can be seen from Figure 6, the microparticles enter from the inlet chamber with 70 V combined driving voltage, and then fill in the inlet chamber in *t* = 15 s, and the velocity increases when the particles go through the narrow diffuser microchannel on the left in Figure 5i. The velocity decreases after the particles enter the center pump chamber due to the increase of the chamber volume, and concentrate in the middle line in Figure 5j when *t* = 150 s. Finally, the particles flow out from the right nozzle microchannel. The whole flow of the solution from the inlet to outlet takes 400 s in Figure 5k. The order of instant velocity of the microparticles in Figure 5i–k is the same as that in Figure 5b–g.

### 3.2. Experiment Results Discussion

The flow rate results of the micropump after testing are shown in Figure 6. Figure 6a,b show the results with single LC driving and single PZT under three voltages, respectively. It can be seen that the average flow rate (the net flow rate affected by the diffuser/nozzle structure) is 0.1316 μL/min, 0.2454 μL/min and 0.4457 μL/min when the voltage is 30 V, 50 V, and 70 V, respectively, in Figure 6a. Therefore, the higher the driving voltage, the larger of flow rate in both single driving modes in Figure 6a,b. That is, when using single actuation, the amount of pumping liquid crystal per unit time increases with the increase of the driving voltage.

However, the flow rate of the micropump increase, and then decrease along with the increase of the driving frequency under single PZT driving in Figure 6b. There is a maximum flow rate value in the whole driving frequency. That means the driving frequency has important influence on the flow rate. Moreover, the frequency point corresponding to the maximum flow moves backward with the increase of voltage. At 30 Vpp, the best frequency is near 30 Hz, and the corresponding maximum flow rate is 1.6681 μL/min. When the voltage is 50 Vpp, the optimum frequency is near 50 Hz, the corresponding maximum flow rate is 1.9055 μL/min. When the voltage is 70 Vpp, the best frequency is near 100 Hz, and the corresponding maximum flow rate is 3.8073 μL/min. When the frequency is 350 Hz, the flow rate exhibits a second peak, but the value is much less than the first one under all three driving voltages. That means there is a second resonance frequency around 350 Hz for the PZT.

Figure 6c is the comparison of the flow rate between combined driving method and single PZT driving method. Since there is no frequency for the DC voltage, just the 70 V voltage is used for fixed LC driving under the combined driving mode. The AC voltages are the same as the single PZT driving mode for ease of comparison. It can be seen that the changing trend of the flow rate along with the frequency in combined driving mode is basically the same as that of the single piezoelectric driving mode. That means the PZT driving has the leading role with respect to the micropump performance. However, by comparing Figure 6b,c, we find that the value of the flow rate is higher under combined driving than that of single PZT driving with the same driving frequency. Furthermore, compared with Figure 6a,c, it is found that the flow value is larger than the single LC driving under 70 V. These results validate our hypothesis in the first part of the study and the numerical analysis parts. Additionally, the maximum flow rate is 4.4941 μL/min when the driving voltage and frequency are 70 Vpp, 100 Hz, and 70 V under combined driving mode. The power consumption under three driving mode are too small, that can be ignored because of the lower current (the current range is from 1 mA to 2 mA) with DC and AC power supplies.

**Figure 7:**
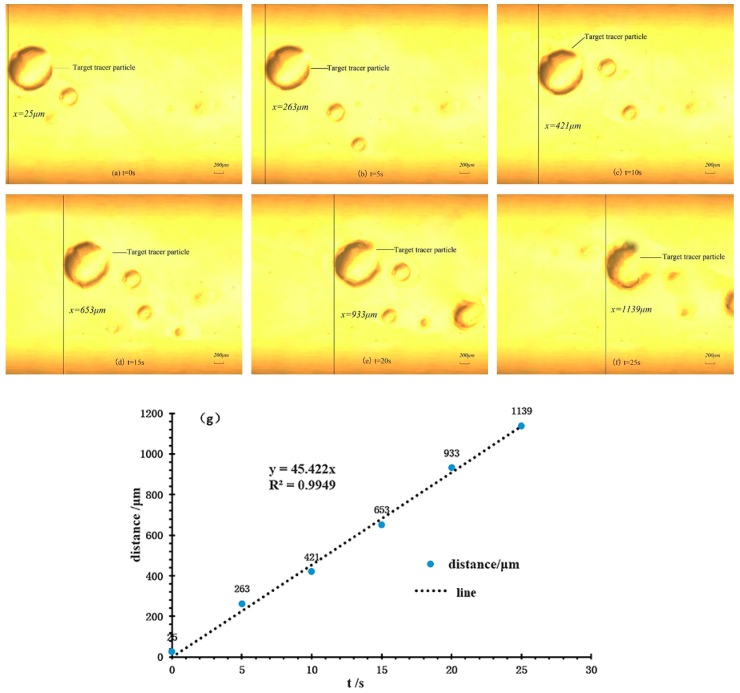
The motion of the LC molecule according to the testing under PZT of 70 Vpp, 100 Hz voltage and LC driving at 70 V. (**a**–**f**) The motion of PS particles in the flow field. (**g**) The curve of the LC molecule motion vs. time.

Figure 7a–f shows the results of flow field in the combined driving mode of 70 Vpp, 100 Hz, and 70 V for AC and DC voltages, respectively. The flow trajectory of the marked PS particles move with the liquid crystal flow in 25 s. The coordinate set of the image region is shown as follows: the lower left corner of the screen is the coordinate origin, the right and upper side is the positive direction of the X axis and Y axis during observing, respectively. The PS particles moved from 25 µm to 1139 µm along with the positive direction of the X axis in 25 s in Figure 7a–f. In order to find out the relationship between the moving distances of tracer particles and time more accurately, the linear equation is obtained by using the data analysis function in Matlab (The MathWorks, Inc., Natick, MA, USA).

The linear correlation coefficient $R^{2}$ is 0.9949 in Equation (21). It can be seen that the linear equation can accurately describe the linear relationship between the moving distance and time of PS particles, as shown in Figure 7g.

(21) y=45.422x

According to the continuity equation of steady flow with incompressible fluid, and concern the practice situation in this study, the flow rate of the micropump can be calculated as follows:

(22) $$Q=V\times A=\frac{\pi d^{2}l}{4t}$$

where *A* is the cross-section area of the outlet pipe, *d* is the diameter of the pipe, the value is 1.5 mm.

The average flow rate of the liquid crystal flow is 4.8160 μL/min according Equation (22). This result is in good agreement with the flow rate experimental results (4.4941 μL/min) of the liquid crystal in the combined driving mode under the same conditions in Figure 6. Additionally, this value is larger than the report in the literature [34] with single LC driving.

## 4. Conclusions

In this paper, a new PZT/LC microfluidic combined actuation scheme is proposed firstly, and the driving mechanism of the piezoelectric effect and liquid crystal backflow effect under the combined driving mode are analyzed in detail. The multi-field coupling equations between electrical, mechanical, and fluid are constructed and refined. The numerical analysis of the multifield coupling is conducted according the equations set. Finally, the sandwiched structural pump body is fabricated by glass wet etching, and the whole micropump is packaged by irreversible connection method with PDMS. The flow field test platform of single and combined driving method is built.

Through the multi-field coupling numerical analysis and experimental analysis, it is found that the combined driving mode realizes “one plus one becomes more than two”. That is, the pumping capacity of the micropump with the combined PZT/LC driving mode is much larger that of the single PZT or LC driving modes. Moreover, the experimental result is consistent with the numerical analysis results. The maximum flow rate of the liquid crystal reaches 4.4941 μL/min under 70 Vpp, 100 Hz of AC voltage, and 70 V of DC voltage according to the flow rate test. The PS particle trajectory is studied in both simulation and test. The actual flow of LC is 4.8160 μL/min according to the linearization equation y=45.422x between the distance and time of the PS particles. This result is consistent with the experimental results of flow rate test of the micropump under the same driving conditions. Through comparison and verification, these velocity values are higher than previously reported literature with single LC driving, and the driving voltage is lower than those in literature with single PZT driving. Overall, the experimental and numerical data are in good agreement, which further demonstrates the feasibility and performance advantages of the novel combined driving method proposed in this paper.

## Figures and Tables

**Table 1:** Construction parameters of the liquid crystal.

Content	Value
Density/ρ	0.85 kg/m^3^
Dynamic viscosity/$\mu_{1}$	32 Pa·s
Rotational viscosity/$\mu_{2}$	229
Temperature/*T*	300 K
Electric permittivity/$\varepsilon_{∥}$ and $\varepsilon_{⊥}$	$15.7\times 10^{11}\;F/m$, $5.7\times 10^{11}\;F/m$
Refractive index/$n_{∥}\;\mathrm{and}\;n_{⊥}$	0.12–0.15
Elastic constant/$K_{1}$,$K_{2}\;\mathrm{and}\;K_{3}$	$6.37\times 10^{12}\;N,3.81\times 10^{12}\;N,8.60\times 10^{12}\;N$
Conductivity of the ionic solution/σ	100 s/cm

**Table 2:** Material parameters of the PZT.

Piezoelectric Material	PZT-5H	Copper
Diameters (mm)	9	12
Thickness (mm)	0.05	0.12
Young’s modulus (MPa)	2000	1
Poisson’s ratio	0.3	0.32
$C_{31}$ (pC/N)	–274	
$C_{32}$ (pC/N)	–274	
$C_{33}$ (pC/N)	593	
Density (kg/m^3^)	7500	1150
Electromechanical coupling factor k	0.39	
Relative permittivity	4	
